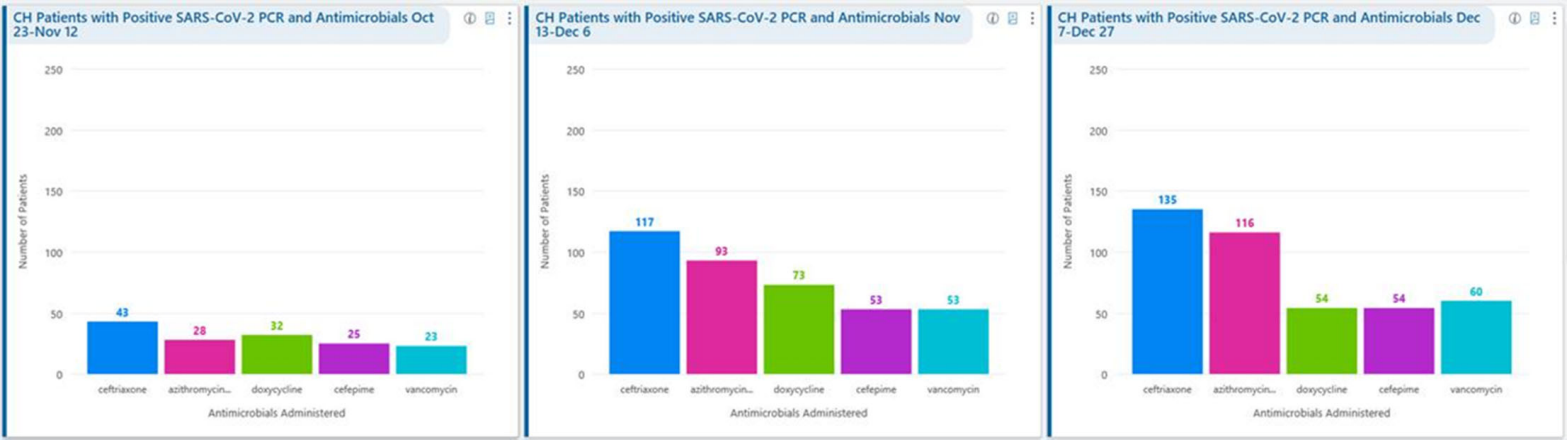# Stewardship of Remdesivir Use in a Rural Community Hospital During the COVID-19 Pandemic

**DOI:** 10.1017/ash.2021.83

**Published:** 2021-07-29

**Authors:** Raghavendra Tirupathi, Melissa Gross

## Abstract

**Background:** Remdesivir was granted EUA followed by full FDA approval for treatment of hospitalized COVID-19 patients on October 22, 2020, based on the results from the ACTT1 trial. Remdesivir use was initially restricted to infectious disease (ID) physicians in our hospital with prescription needing formal ID consultation until complete approval. Due to increasing case counts in our hospital, a decision was made to allow intensivists and hospitalists the authorization to prescribe remdesivir in a phased manner. In this retrospective study, we assessed the impact of phased-in prescribing on remdesivir utilization and days of therapy of antimicrobials. **Methods:** Remdesivir prescribing was streamlined by real-time institutional guidelines developed by a COVID-19 treatment committee constituting ID and other clinicians. Eligibility for remdesivir included positive SARS-CoV-2 PCR test, severe disease defined as persistent hypoxia (<94% oxygen saturation on room air), requiring supplemental oxygen and/or on mechanical ventilation (MV) for <72 hours, and symptom onset of <10 days. We retrospectively reviewed cohorts of 3 periods during which remdesivir was prescribed. In the first cohort A, between October 23, 2020, and November 12, 2020, remdesivir was restricted to ID physicians with formal ID consultation. Cohort B comprised inpatients between November 13, 2020, and December 6, 2020, when hospitalists and intensivists were allowed to prescribe remdesivir through an EMR order set after prior authorization by an ID physician via curbside or telephonic consultation. Cohort C, from December 7, 2020, to December 26, 2020, comprised inpatients with unrestricted prescribing of remdesivir by hospitalists and intensivists. We also evaluated antibiotic use. **Results:** In cohort A, SARS CoV-2 positivity was 20.3%; 64 inpatients tested positive and 35 patients (54.7%) who met the criteria were prescribed remdesivir after a formal consultation with an ID physician. In cohort B, requiring prior authorization by an ID physician, SARS-CoV-2 positivity rapidly increased to 34%; 193 patients tested positive and 97 patients (50.3%) received remdesivir. In cohort C, during unrestricted access, positivity further increased to 38%; 235 inpatients tested positive and 123 (52.5%) received remdesivir. Remdesivir use remained steady during the 3 phases of gradual de-escalation of restricted prescribing and safe handoff in the context of clear guidelines, as well as ongoing curbside education provided by ID physicians during the second phase. Cohort B demonstrated the best prescribing rates. Antimicrobial prescribing data were also collected during the 3 cohort phases (Figures 1–3). **Conclusions:** Remdesivir is an expensive antiviral with limited utility and maximum benefit in COVID-19 inpatients who are hypoxic but do not require mechanical ventilation. Stewardship of remdesivir with safe, gradual handoff to inpatient can be achieved without overuse.

**Funding:** No

**Disclosures:** None

**Figure 1.**
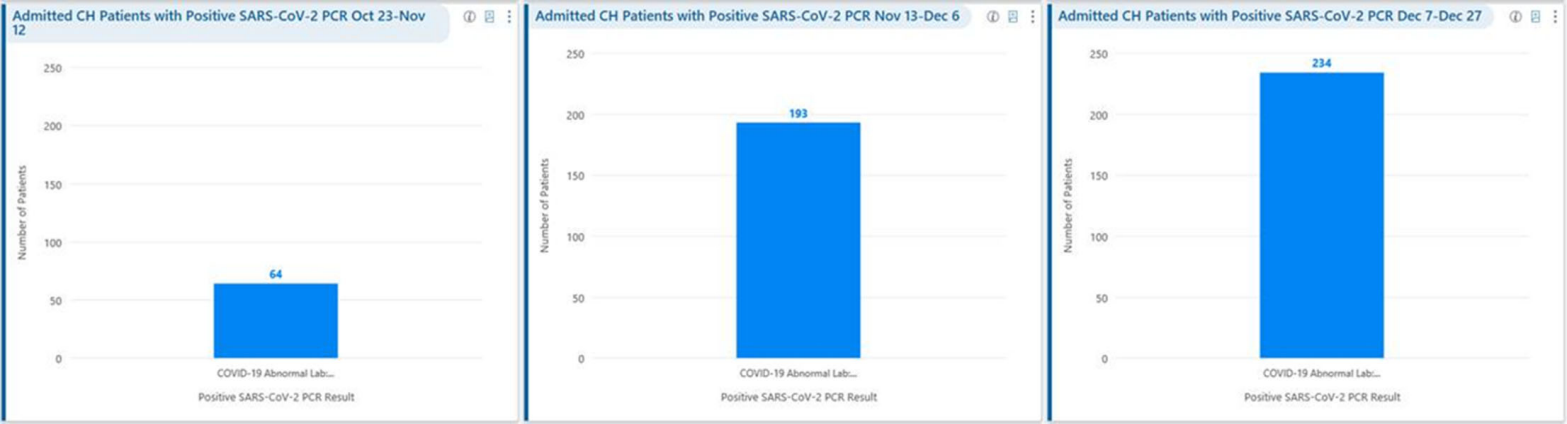

**Figure 2.**
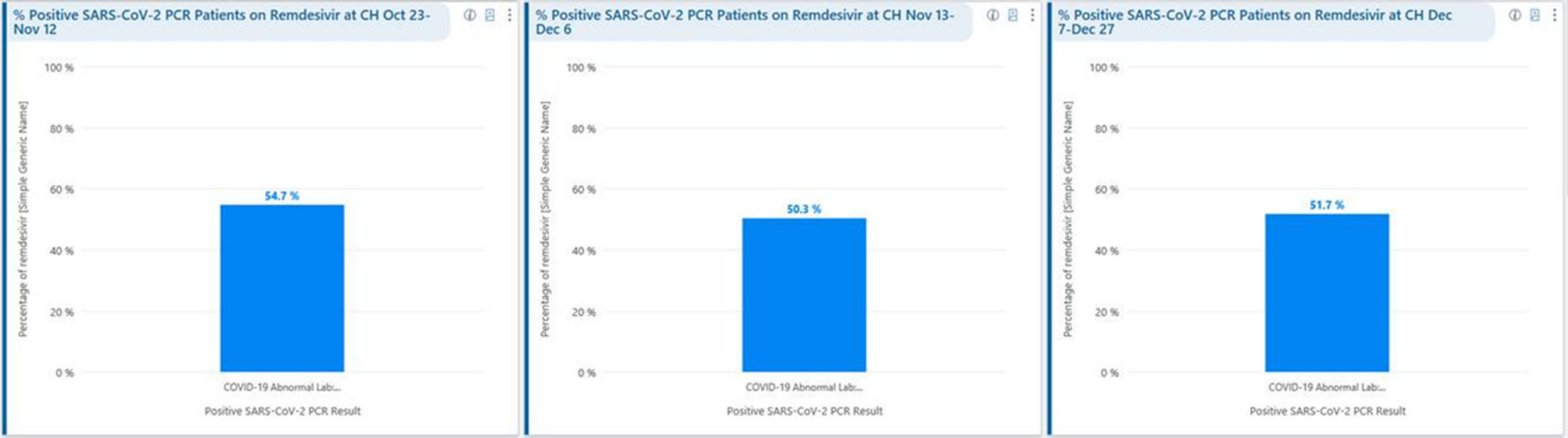

**Figure 3.**